# Patient Perspectives on AI for Mental Health Care: Cross-Sectional Survey Study

**DOI:** 10.2196/58462

**Published:** 2024-09-18

**Authors:** Natalie Benda, Pooja Desai, Zayan Reza, Anna Zheng, Shiveen Kumar, Sarah Harkins, Alison Hermann, Yiye Zhang, Rochelle Joly, Jessica Kim, Jyotishman Pathak, Meghan Reading Turchioe

**Affiliations:** 1 School of Nursing Columbia University New York, NY United States; 2 Department of Biomedical Informatics Columbia University New York, NY United States; 3 Mailman School of Public Health Columbia University New York, NY United States; 4 Stuyvestant High School New York, NY United States; 5 College of Agriculture and Life Science Cornell University Ithaca, NY United States; 6 Department of Psychiatry Weill Cornell Medicine New York, NY United States; 7 Department of Population Health Sciences Weill Cornell Medicine New York, NY United States; 8 Department of Obstetrics and Gynecology Weill Cornell Medicine New York, NY United States

**Keywords:** artificial intelligence, AI, mental health, patient perspectives, patients, public survey, application, applications, health care, health professionals, somatic issues, radiology, perinatal health, Black, professional relationship, patient-health, autonomy, risk, confidentiality, machine learning, digital mental health, computing, coding, mobile phone

## Abstract

**Background:**

The application of artificial intelligence (AI) to health and health care is rapidly increasing. Several studies have assessed the attitudes of health professionals, but far fewer studies have explored the perspectives of patients or the general public. Studies investigating patient perspectives have focused on somatic issues, including those related to radiology, perinatal health, and general applications. Patient feedback has been elicited in the development of specific mental health care solutions, but broader perspectives toward AI for mental health care have been underexplored.

**Objective:**

This study aims to understand public perceptions regarding potential benefits of AI, concerns about AI, comfort with AI accomplishing various tasks, and values related to AI, all pertaining to mental health care.

**Methods:**

We conducted a 1-time cross-sectional survey with a nationally representative sample of 500 US-based adults. Participants provided structured responses on their perceived benefits, concerns, comfort, and values regarding AI for mental health care. They could also add free-text responses to elaborate on their concerns and values.

**Results:**

A plurality of participants (245/497, 49.3%) believed AI may be beneficial for mental health care, but this perspective differed based on sociodemographic variables (all *P*<.05). Specifically, Black participants (odds ratio [OR] 1.76, 95% CI 1.03-3.05) and those with lower health literacy (OR 2.16, 95% CI 1.29-3.78) perceived AI to be more beneficial, and women (OR 0.68, 95% CI 0.46-0.99) perceived AI to be less beneficial. Participants endorsed concerns about accuracy, possible unintended consequences such as misdiagnosis, the confidentiality of their information, and the loss of connection with their health professional when AI is used for mental health care. A majority of participants (80.4%, 402/500) valued being able to understand individual factors driving their risk, confidentiality, and autonomy as it pertained to the use of AI for their mental health. When asked who was responsible for the misdiagnosis of mental health conditions using AI, 81.6% (408/500) of participants found the health professional to be responsible. Qualitative results revealed similar concerns related to the accuracy of AI and how its use may impact the confidentiality of patients’ information.

**Conclusions:**

Future work involving the use of AI for mental health care should investigate strategies for conveying the level of AI’s accuracy, factors that drive patients’ mental health risks, and how data are used confidentially so that patients can determine with their health professionals when AI may be beneficial. It will also be important in a mental health care context to ensure the patient–health professional relationship is preserved when AI is used.

## Introduction

### Background

The potential of artificial intelligence (AI) to transform health care has been touted since the early 2010s [1-4]. In health care applications, practitioners commonly operationalize AI by training machine learning algorithms using large retrospective data sets to perform human reasoning tasks, such as identifying issues (eg, anomalies in medical images), predicting events (eg, disease incidence), recommending treatments (eg, pharmacogenomics), detecting patterns (eg, finding symptom clusters), and generating text (eg, for clinical decision support rules).

AI has already made significant strides in the field of medical imaging, aiding health professionals at various stages, including improving image quality [5], guiding image acquisition [6], risk-stratifying images to be reviewed by a specialist (ie, a radiologist) [7,8], and interpreting images [9,10]. More recently, predictive AI has been leveraged to detect mental health–related issues, including major depressive disorders [11,12], stress, anxiety [13], bipolar disorder [14], and even suicide [15,16]. AI has also commonly been used for treatment selection in fields such as oncology and mental health [17,18]. Despite the demonstrated predictive accuracy of AI, relatively few of the predictive AI tools created are implemented in everyday clinical care [19], and even fewer tools have demonstrated a positive clinical impact compared to current standards of care [20-22]. Furthermore, of the 89 unique articles in 2 systematic reviews of clinical trials evaluating predictive AI, none focused on mental health–related conditions [20,21].

Due to the gap between the predictive AI’s accuracy and its lack of observed impact on health outcomes, many researchers in many countries have studied health professionals’ perceptions of AI-based tools and related implementation challenges [16,23-28]. However, patients’ perspectives of AI have been understudied [29-31]. While some predictive AI developers may not intend for patients to view the AI’s output on their own, it has become more likely that patients have access to predictive AI output due to recent advances in patient data ownership and access. The US 21st Century Cures Act, for example, prevents blocking information from patients, requiring health organizations and insurance providers to give patients access to their eHealth information without delay or expense [32]. This may result in a patient seeing a predictive AI risk score before a discussion with their health care team. In a 2020 predictive AI preimplementation study, health professionals stressed the importance of keeping the patient in the information loop when the AI predicts a risk or recommends a treatment to justify to the patient why they may require further support [24]. In addition to practical considerations, there is an ethical imperative to ensure patients understand how their data are being used, what predictive AI may reveal, and what the insight means, especially for sensitive issues, such as mental health care concerns [33]. Before we design solutions for communicating AI information to patients, it is important to understand the public’s perceived benefits, comfort, concerns, and values related to AI use, particularly for mental health care [34].

To address the deficit in knowledge regarding patient perspectives on AI, Khullar et al [35] conducted a survey of a nationally representative panel of the US-based population. While most respondents reported a perceived benefit of using AI in health care, comfort with AI unsurprisingly varied based on the accuracy, transparency, and clinical application of AI (eg, reading a chest x-ray vs making a cancer diagnosis) [35]. The survey conducted by Khullar et al [35] focused on somatic applications of AI, leaving questions regarding public perceptions of AI for mental health care applications unanswered. However, others have explored narrower issues related to feedback on specific mental health care apps and specific prediction tasks (eg, the prediction of suicide) [36,37].

### Objectives

AI applications for mental health are rapidly increasing as patients gain greater access and ownership of their data. Given the ethical concerns regarding the creation and use of AI and the stigmas surrounding mental health care, understanding patients’ perceptions of whether and how AI may be appropriately used for mental health care is critical [38,39]. This study adapts and extends the survey conducted by Khullar et al [35] to evaluate patient perspectives on the use of AI for mental health care applications. We specifically surveyed members of the public to gain patient perspectives on AI applications for mental health. Khulllar et al [35] did not explore values regarding AI use, that is, what patients’ priorities for effective, appropriate AI use for mental health care are. We also explored these values in this study using a bioethics-informed framework. The specific research questions (RQs) guiding our work were as follows:

- RQ 1: Do the public perceive AI to be beneficial for mental health care? RQ 1 Equity: Do perceived benefits differ by sociodemographic factors?
- RQ 2: How concerned is the public about common issues related to AI use in mental health care?
- RQ 3: What types of predictive tasks are the public comfortable with AI executing in mental health care applications?
- RQ 4: What are the public’s values related to AI use for mental health care?

We also elicited open-ended responses from participants to add to their quantitative feedback.

## Methods

### Study Design

In our study, we conducted a 1-time, cross-sectional survey of US-based adults in September 2022. We sampled a general US adult population to elicit the public’s perspectives on AI for mental health. We partnered with Prolific (Prolific Academic Ltd), a web-based survey sampling platform, to recruit participants. Prolific provides access to an international sample of verified users (>100,000 users residing in the United States) who are willing to be involved in survey research studies. Prolific matches eligible participants with research studies, streamlining the recruitment, data collection, and compensation processes. Prospective participants had to be verified Prolific users aged ≥18 years who were fluent in written and spoken English to be eligible.

### Ethical Considerations

This study was approved by the BRANY Institutional Review Board (protocol 22-01024358). Before answering the questionnaire, participants read an information sheet and consented to participate in this specific study. Participants who completed the full questionnaire were compensated at an hourly rate of US $13.60 based on Prolific’s policies.

### Questionnaire Design

We designed our questionnaire such that it mimicked items asked in a previous study by Khullar et al [35] but applied to perspectives specifically related to AI for mental health, instead of AI for health care broadly. Question categories related to AI for mental health care were as follows: (1) perceived benefits of AI, (2) concerns about AI, and (3) comfort with using AI for specific predictive tasks. Adapting questions related to perceived benefits and concerns predominantly involved updating the terms “health” or “health care” to “mental health” or “mental health care,” respectively. In the questionnaire developed by Khullar et al [35], questions regarding predictive tasks included those on reading a screening tool (ie, a chest x-ray); making a diagnosis for 2 different conditions, with 1 being more severe (pneumonia and cancer); and telling a patient they had either of the 2 aforementioned conditions and making a treatment recommendation. Our team worked with a trained psychiatrist to construct tasks following similar patterns but pertaining to mental health care, adding 2 more tasks (resulting in a total of 7 tasks) to explore more sensitive concepts relating to mental health. Multimedia Appendix 1 presents the questions in each of the aforementioned categories along with the question from which they were adapted as applicable.

We also extended the questionnaire to understand participant’s values pertaining to AI design and implementation for mental health care to facilitate a more patient-centered design of future AI applications for mental health. This section asked patients to rate their level of importance regarding various statements pertaining to AI for mental health care, as informed by the constructs of MITRE’s bioethical framework. Multimedia Appendix 1 displays the value statements presented to participants based on the relevant bioethics constructs.

In addition to the questions on perspectives and values, participants also answered questions on sociodemographic characteristics, including personal characteristics, health literacy, subjective numeracy, previous mental health care experience, and pregnancy history (results reported in a separate manuscript). The full battery of sociodemographic questions is presented in Multimedia Appendix 1.

Finally, following the sections regarding concerns and values, the survey contained open-ended questions to allow people to provide free-text responses with additional concerns or values.

We designed the battery of questions with input from experts in AI, human-centered design, and psychiatry and the author of the original survey from which the questions were adapted. The survey questions underwent 2 rounds of pilot testing to improve their comprehensibility and understand the amount of time needed to complete the questionnaire. The question-and-answer design was optimized and pilot tested for completion on both desktops and mobiles (ie, smartphones) to ensure those with different devices or preferences could participate in the study.

### Participants

All participants were recruited from Prolific’s survey sampling panel and were verified users who agreed to participate in research studies via the Prolific website. Our sample included those aged ≥18 years, residing in the United States, and with the ability to speak and read English. We recruited a sample representative of the adult US population in terms of age, race, and gender, according to the US Census. We initially recruited 530 survey respondents, of whom 30 (5.7%) did not begin the survey after reading the informed consent document, resulting in a total of 500 (94.3%) respondents. All 500 respondents finished the survey (0 incomplete responses) over a median time of 15 minutes and 24 seconds.

### Data Collection

Our team designed and programmed the questionnaire using the Qualtrics XM (Qualtrics) platform. Participants received an invitation to complete the questionnaire through Prolific and then proceeded by clicking on a secure, anonymous link to Qualtrics. Participants could complete the survey using any smartphone, tablet, or computer, provided they had an internet connection. Participants then completed the questionnaire. There was no time limit for completing the questionnaire, and participants had the option to pause and resume completing the questionnaire at a later time. Participants also had the option to discontinue the survey at any time.

### Data Analysis

#### Quantitative Analysis

The first level of analysis involved assessing descriptive statistics to understand trends in participant perceptions and values. We also selected an outcome of interest (perceived benefit of AI for mental health) and created a logistic regression model to better understand whether perceived benefits may differ by sociodemographic factors, specifically age, gender, race, education, financial resources, mental health history, and self-rated health literacy [40]. The α value for all analyses was set at .05, and the R software (version 4.2.1; The R Foundation) was used. An analysis of a subset of this data (only those of participants reporting female sex at birth) related to differences in perspectives based on pregnancy history has been reported in a separate manuscript [41].

#### Qualitative Analysis

We analyzed free-text responses through an inductive thematic analysis and a constant comparative process. One analyst initially reviewed the codes and created a draft codebook. Free-text responses to the 2 open-ended questions were analyzed using a singular coding scheme. A second analyst then used the coding scheme to independently dual code each free-text response. The analysts met with a third team member to resolve discrepancies, coding via consensus and updating the codebook throughout the discussion. Once detailed codes had been developed and 50% of the initial coding was completed, the team completed axial coding, coming up with higher-level summary themes to describe patterns in the detailed codes.

## Results

### Participant Characteristics

Table 1 describes the demographic makeup of the 500 adult, US-based survey respondents sampled using the Prolific platform [42]. Respondents were nationally representative based on race, age, and gender.

**Table 1:** Participant demographics (N=500).

Participant characteristics		Values
**Age (y)**		
	Median (IQR)	46 (31-59)
	Mean (SD; range)	46 (16; 18-93)
**Gender, n (%)**		
	Women	249 (49.8)
	Men	238 (47.6)
	Transgender	1 (0.2)
	Something else	9 (1.8)
	Prefer not to answer	3 (0.6)
**Race, n (%)**		
	Asian	25 (5)
	Black or African American	66 (13.2)
	White	388 (77.6)
	Other or prefer not to answer^a^	21 (4.2)
**Perceived financial resources, n (%)**		
	More than enough	65 (13)
	Enough	271 (54.2)
	Not enough	156 (31.2)
	Prefer not to answer	8 (1.6)
**Mental health history^b^, n (%)**		
	Yes	215 (43)
	No	271 (54.2)
	Prefer not to answer	14 (2.8)
**Health literacy^c^, n (%)**		
	Adequate	369 (73.8)
	Inadequate	131 (26.2)

^a^Answer options included American Indian or Alaskan Native, Native Hawaiian or Pacific Islander, and prefer not to answer, and multiple options could be selected.

^b^The question asked was “Have you ever been told that you have mental illness?”

^c^Measured using the Brief Health Literacy Screener developed by Chew et al [40].

### RQ 1: Perceived Benefits of AI for Mental Health

Participants were first asked, “Overall, in the next 5 years, do you think AI will make mental health care in the United States...” Answer options included “much better,” “somewhat better,” “minimal change,” “somewhat worse,” “much worse,” and “don’t know.” We computed a logistic regression model such that “much better” and “somewhat better” were classified as 1, and the other responses were classified as 0, excluding the 3 (0.6) participants, among the total 500 participants, answering, “don’t know.” Among 497 included respondents, 245 (49.3%) respondents believed that AI would make mental health care better or much better. Table 2 reveals that participants of Black or African American race (*P*=.04; odds ratio [OR] 1.76, 95% CI 1.03-3.05) and those with lower health literacy (*P*=.004; OR 2.19, 95% CI 1.29-3.78) were significantly more likely to endorse that AI would make mental health care somewhat or much better. Women, by contrast, were significantly less likely to endorse this statement (*P*=.046; OR 0.68, 95% CI 0.46-0.99).

**Table 2:** Logistic regression results: impact of sociodemographic variables on the perceived benefit of artificial intelligence (AI) for mental health.

Variable	β estimate	Odds ratio (95% CI)	*P* value
Intercept	0.378	—^a^	.37
Age (y)	–0.006	0.99 (0.98-1.01)	.36
Gender (woman)	–0.388	0.68 (0.46-0.99)	.046^b^
Race (Black or African American)	0.567	1.76 (1.03-3.05)	.04^b^
Perceived financial resources (not enough)	0.003	1.00 (0.66-1.51)	.99
Mental illness history (yes)	–0.044	0.96 (0.65-1.42)	.83
Health literacy (inadequate)	0.782	2.19 (1.29-3.78)	.004^b^

^a^Not available.

^b^Statistically significant based on α<.05.

### Concerns, Comfort With Predictive Tasks, and Values (Quantitative)

#### RQ 2: Concerns Regarding AI for Mental Health Care

**Figure 1:**
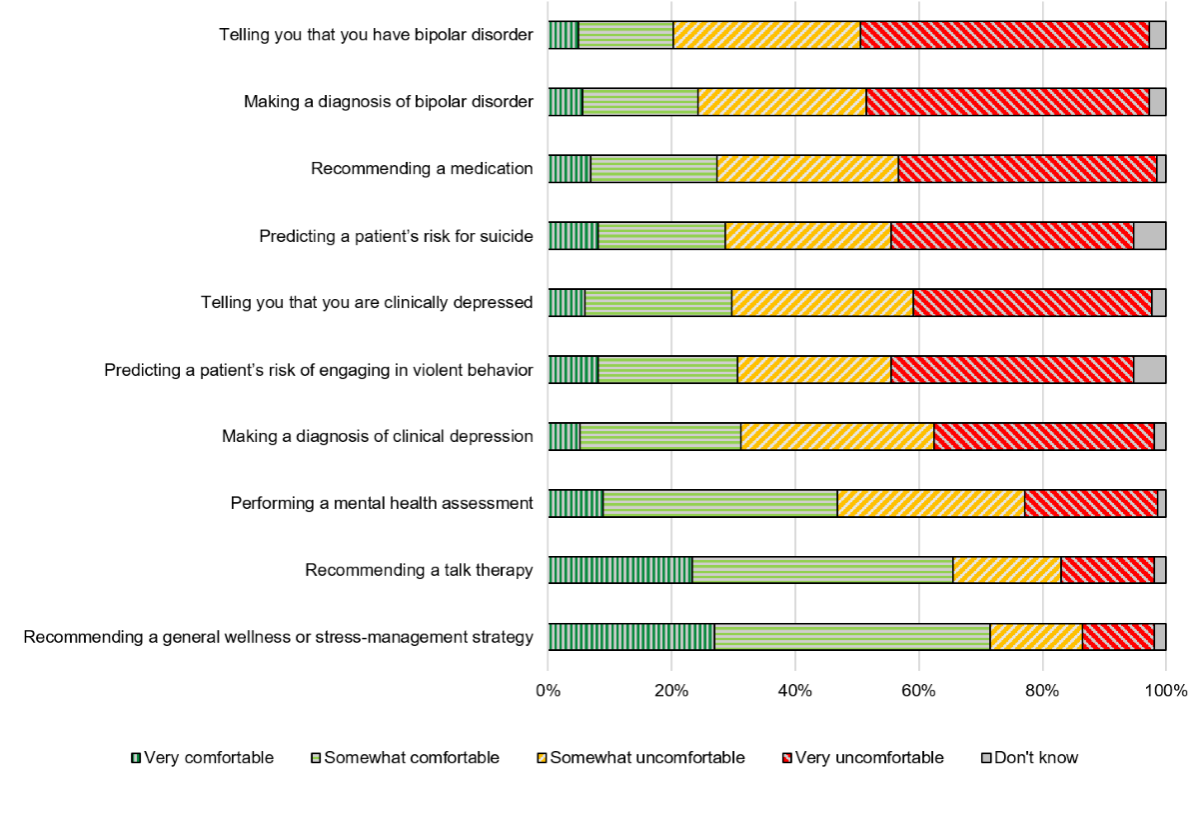
Reported levels of perceived concerns regarding artificial intelligence (AI) use for mental health.

On the basis of the survey conducted by Khullar et al [35], we asked participants their level of concern (very concerned, somewhat concerned, not concerned, and don’t know) related to 6 potential challenges of using AI for mental health care (Figure 1). Participants reported being somewhat or very concerned about AI making the wrong diagnosis (402/500, 80.4%), leading to inappropriate treatment (435/500, 87%), or leading to them not knowing their mental health care provider well (409/500, 81.8%). Participants reported being very or somewhat concerned regarding spending less time with their mental health care professional (346/500, 69.2%) and their confidentiality (302/500, 60.4%) but expressed relatively less concern regarding increased costs (217/500, 43.4%).

#### RQ 3: Comfort With AI Accomplishing Mental Health Care Tasks

**Figure 2:**
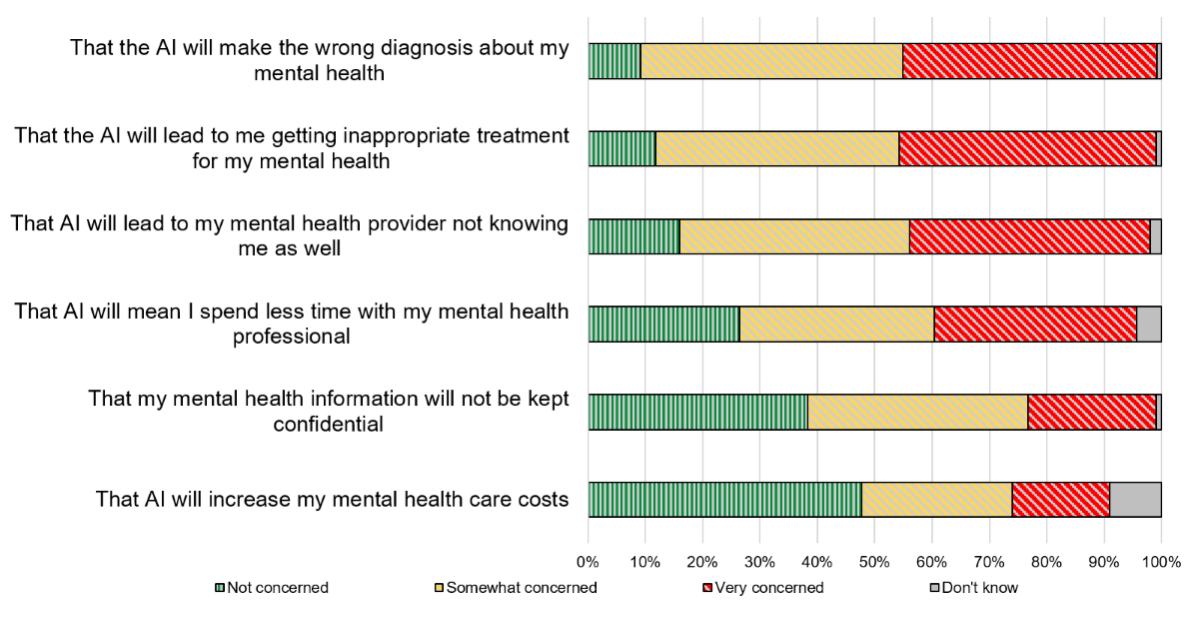
Reported level of comfort with artificial intelligence (AI), instead of a mental health professional, conducting various tasks.

Next, we asked patients their level of comfort with AI performing various tasks instead of their mental health care professional. We assessed a range of tasks (ie, assessment, diagnosis, diagnosis delivery, and treatment recommendation) and mental health issues of varied levels of perceived severity (ie, depression, bipolar disorder, and suicide), as shown in Figure 2. Participants were the most comfortable (reporting being very or somewhat comfortable) with recommendations of nonpharmacological interventions, including general wellness management strategies (357/500, 71.4%) and talk therapy (328/500, 65.6%). Participants expressed moderate comfort with AI performing a mental health care assessment but were less comfortable (with only 20% to 32% selecting very or somewhat comfortable) with various prediction, diagnosis, and diagnosis delivery tasks, as well as a medication recommendation task. Participants were the least comfortable with diagnosis delivery tasks (ie, telling someone directly they have a mental health condition), including for clinical depression (123/500, 24.6%) and bipolar disorder (103/500, 20.6%).

We also asked participants their level of comfort sharing mental health information with a (human) mental health care professional, an AI chatbot, or an AI program that treats disease to improve it. We chose these categories to understand perspectives of common uses of patient data for AI, such as the use of patient data to build models that make predictions or help treat diseases as compared to the use of patient information directly for patient support (eg, an AI chatbot). Patients were the most comfortable (very or somewhat) sharing information with a mental health care professional (389/500, 77.8%), followed by sharing with an AI program that treats disease (300/500, 60%) and then sharing with an AI chatbot (238/500, 47.6%).

#### RQ 4: Values Related to AI for Mental Health

#### Overview

We replicated a series of questions from Khullar et al [35] assessing patient values of various aspects of AI, including transparency, explainability and performance, responsibility, and the effect of AI on trust in health professionals (Table 3).

**Table 3:** Summary of questions regarding values related to artificial intelligence (AI) for mental health (N=500).

Value, question, and answer choice				Respondents, n (%)
**Transparency of AI use**				
	**How important do you think it is that you are told when an AI program has played a big role in your mental health diagnosis or treatment?**			
		Not important	13 (2.6)	
		Somewhat important	111 (22.2)	
		Very important	364 (72.8)	
		Don’t know	12 (2.4)	
	**How important do you think it is that you are told when an AI program has played a small role in your mental health diagnosis or treatment?**			
		Not important	39 (7.8)	
		Somewhat important	128 (25.6)	
		Very important	253 (50.6)	
		Don’t know	10 (2)	
	**[Clinical scenario]^a^ How important is it that your doctor tells you that the computer program helped make this decision?**			
		Not important	25 (5)	
		Somewhat important	66 (13.2)	
		Very important	388 (77.6)	
		Don’t know	21 (4.2)	
**Explainability and performance**				
	**How comfortable would you be receiving a mental health diagnosis from a computer program that made the right diagnosis 90%** **of the time but could not explain why it made the diagnosis?**			
		Very comfortable	15 (3)	
		Somewhat comfortable	94 (18.8)	
		Somewhat uncomfortable	184 (36.8)	
		Very uncomfortable	199 (39.8)	
		Don’t know	8 (1.6)	
	**How comfortable would you be receiving a mental health diagnosis from a computer program that made the right diagnosis 98% of the time but could not explain why it made the diagnosis?**			
		Very comfortable	63 (12.6)	
		Somewhat comfortable	138 (27.6)	
		Somewhat uncomfortable	172 (34.4)	
		Very uncomfortable	117 (23.4)	
		Very comfortable	10 (2)	
		Don’t know	63 (12.6)	
**Responsibility and AI**				
	**Imagine that your mental health professional and a computer program work together to treat your mental illness and a medical error occurs. An example of a medical error is getting a diagnosis that was wrong, or a treatment that was not needed. Who is responsible? (Select all that apply.)^b^**			
		The mental health professional	412 (82.4)	
		The company that made the computer program	30 (6)	
		The hospital or clinic that bought the computer program	20 (4)	
		The government agency that approved the computer program	6 (1.2)	
		Someone else	12 (2.4)	
		No one	1 (0.2)	
		Don’t know	19 (3.8)	
	**Imagine that you have a sleeping disorder that might be due to a mental health issue. You have a test done. Your doctor uses a computer program that says the sleeping disorder might be mental health-related, so you start medication to treat it. The medication leads to bad side effects. After another doctor evaluates your sleeping disorder, it turns out it was NOT mental health related. Who, if anyone, is to blame? (Select all that apply.) ^b^**			
		The mental health professional	408 (81.6)	
		The company that made the computer program	35 (7)	
		The hospital or clinic that bought the computer program	14 (2.8)	
		The government agency that approved the computer program	5 (1)	
		Someone else	6 (1.2)	
		No one	19 (3.8)	
		Don’t know	13 (2.6)	
	**Imagine that your hospital recently started using a computer program to help diagnose mental health problems. Who do you think has checked to make sure the computer program is safe before it is rolled out? (Select all that apply.)^b^**			
		The mental health professional	135 (27)	
		The company that made the computer program	229 (45.8)	
		The hospital or clinic that bought the computer program	73 (14.6)	
		The government agency that approved the computer program	31 (6.2)	
		Someone else	4 (0.8)	
		No one	16 (3.2)	
		Don’t know	12 (2.4)	
**Effect of AI on trust in mental health professionals**				
	**Imagine that you have some symptoms that have been bothering you for a while, such as difficulty sleeping, eating, and focusing on work. You visit a doctor who runs some tests and he says he does NOT think you have any mental health issue. He also puts your symptoms into a computer program that can make the right diagnosis about 80% of the time, but can’t say why it chose the diagnoses. It says you DO have mental health issue. How does the computer program affect your view?**			
		It would not affect my trust of the mental health professional’s assessment	85 (17)	
		It would make me question the mental health professional’s assessment	265 (53)	
		I do not know if it would change my view of the mental health professional’s assessment.	137 (27.4)	
		Don’t know	13 (2.6)	

^a^Scenario wording: “Imagine that you have been told that you have been diagnosed with depression, a common mental illness that affects your mood, thoughts, and behavior. In the past, your doctor would have decided whether to prescribe a medication or refer you for psychotherapy depending on the type of symptoms you have and how severe they are. //Your doctor now has a computer program that uses many other factors. This computer program says you should start an antidepressant.”

^b^Multiple selections were allowed, so the sum of proportions can be >100%.

#### Transparency of AI Use

Participants were first asked how important it was to know when AI played a (1) small or (2) big role in their mental health treatment or diagnosis. Most participants found it somewhat or very important to know whether AI played a small (450/500, 90%) or big (474/500, 94.8%) role in their mental health treatment or diagnosis, although participants tended to report it was very important based on whether AI played a big (365/500, 73%) versus small (253/500, 50.6%) role. This pattern remained consistent with a specific scenario regarding the use of an AI program in prescribing antidepressants, with many participants stating it was very important (355/500, 71%) or somewhat important (114/500, 22.8%) that their mental health care professional informed them regarding the AI’s involvement in this prescribing decision.

#### Explainability and Performance

In comparing AI that was not explainable (ie, it could not describe why it made a given diagnosis), participants were generally uncomfortable even with stated AI performance accuracies of 90% (383/500, 76.6% somewhat or very uncomfortable) and 98% (289/500, 57.8% somewhat or very uncomfortable).

#### Responsibility and AI

Participants answered a series of questions regarding who was responsible in the event of a medical error when AI was used in conjunction with their mental health treatment; answer options included the following: the mental health professional who made the decision, the company that made the computer program, the hospital or clinic that bought the computer program, the government agency that approved the computer program, someone else, no one, and don’t know. In the case where AI was used in collaboration with a single mental health professional, most participants (>80%) reported that the mental health professional would be the one responsible if a medical error (eg, wrong diagnosis or unnecessary treatment) occurred for both a specific (ie, sleep disorder) and a general scenario.

Participants were more divided on who had responsibility for ensuring an AI program for mental health care was safe, with the plurality stating the company that created the program was responsible (229/500, 45.8%), followed by the mental health professional (135/500, 27%) and then the health system (73/500, 14.6%), with fewer than 10% of the participants selecting each of the remaining answer options.

#### Effect of AI on Trust in Mental Health Care Professionals

Most participants (265/500, 53%) said that if an AI program that was accurate 80% of the time in detecting health issues related to sleeping, eating, and concentrating disagreed with their mental health professional, it would make them question the health professional’s assessment. Notably, nearly 30% (137/500) of participants said they “did not know” how such information would change their view of their mental health professional’s assessment, with the remaining 17% (85/500) stating it would not change.

**Figure 3:**
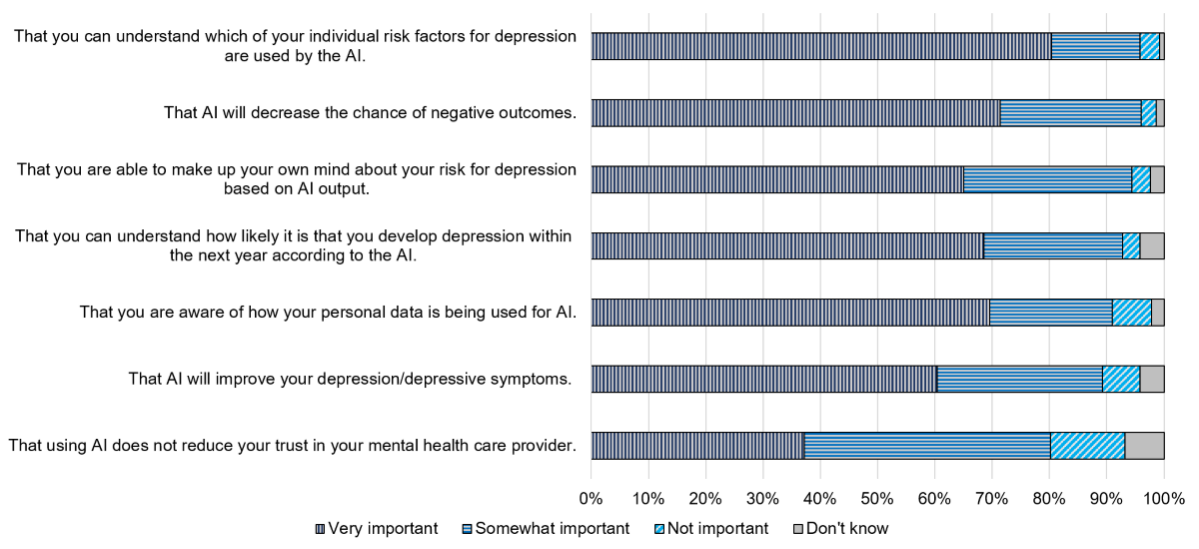
Importance of various values related to artificial intelligence (AI) use for mental health.

To better understand what participants valued the most related to AI for their mental health, we asked the importance of various ethical constructs, based on MITRE’s ethical framework for consumer-generated health information [43], as they pertained to an example AI program used to support treatment for depression (Figure 3). Over 80% (range 80.2%-96%) of participants found each of the constructs somewhat or very important. Notably, the highest proportion of participants (402/500, 80.3%) viewed explainability and transparency, “explainability,” to be very important. Participants tended to perceive decreasing the risk of negative outcomes as slightly more important than improving symptoms. Participants found AI *not* reducing trust in their mental health professional as the least important trait by comparison, although 37.2% (186/500) and 43% (215/500) rated this trait as very and somewhat important, respectively.

### Qualitative Results

Participants provided free-text responses describing themes related to nuanced aspects of AI’s performance, human-AI dynamics, and further values or concerns pertaining to AI. Free-text responses were mandatory, but some participants simply stated they had no additional concerns (165/500, 33%), or they did not provide sufficient detail for their responses to be categorized (7/500, 1.4%). Of the 1000 responses (2 per participant), 97 involved >1 code, so percentages listed subsequently reflect the proportion of total codes observed. On the basis of the results listed in Table 1 (sociodemographic differences in the perceived benefits of AI for mental health care), quotes shown also provide the patients’ gender, race, and self-rated literacy for context.

### AI Performance

Table 4 provides the detailed codes, proportion of occurrence, and examples related to AI performance. Participants described issues related to AI’s accuracy, biases in AI data or biases that may occur in the use of AI, potential errors AI may commit, and how these errors may affect the quality of care. Participants expressed mixed opinions regarding whether AI would improve or degrade the quality of mental health care.

**Table 4:** Detailed qualitative codes related to artificial intelligence (AI) performance.

Detailed code	Count (n=188, 17.9%), n (%)^a^	Example quotes
AI performance and error: accuracy	96 (51.1)	“I think the main concern would be potential for getting an inaccurate diagnosis or the wrong treatment. It would definitely take time to trust the reliability. Programming errors, for example, could potentially lead to fatal outcomes for patients” (White woman with self-rated adequate health literacy).
Bias in data and use	50 (26.6)	“I am concerned that the algorithms/data set that was utilized to train AI would be biased. For instance, if more white people seek mental health care, and AI is trained on their data, would AI be as good at diagnosing mental health conditions in people of color?” “It will be biased, sexist and racist. It will rely on old ideas of mental health care and not use current information. It will be used to ignore or bully patients” (White nonbinary individual with self-reported inadequate health literacy).
Risk of harm	29 (15.4)	“I think there is far too much on the line when it comes to mental health that it’s risky to rely on AI for it. I think my main concern would be being over-diagnosed and having to be put in a psychiatric ward. I think that would be horrific” (White woman with self-rated adequate health literacy).
Care quality	13 (6.9)	“I think that it might lessen the quality standard of hired healthcare professionals since the expertise of AI system could become more important” (Black man with self-reported adequate health literacy). “It would improve the quality of work” (White woman with self-rated adequate health literacy).

^a^Percentages do not add to 100%, as “no additional concerns” and “indeterminable” codes are included in the count.

### AI and Humans: Superior, Inferior, or Simply Better Together

Table 5 presents participant feedback related to human-AI dynamics. Participants described worry that AI may not be able to replicate things done by humans (AI capabilities, human reasoning and communication, and the importance of human connection). By contrast, some pointed out ways in which AI may offer advantages to human cognition (AI capabilities). They also provided feedback on how AI and humans may (or may not) work together (human-AI collaboration and overreliance on AI), with many noting that AI should be overseen by humans and not work autonomously in mental health care applications. Finally, a few participants expressed concerns regarding how AI may take away jobs from humans.

**Table 5:** Detailed qualitative codes related to human–artificial intelligence (AI) dynamics.

Detailed code	Count (n=297. 28.3%), n (%)^a^	Example quote
AI capabilities	78 (26.3)	“A software program’s understanding of mental health will never be as nuanced as a real humans. There will always be less common variables that AI systems aren’t programmed to take into account. I am concerned that with AI driven healthcare, patient[s] with more unusual backgrounds, experiences and symptoms will not have access to human professional[s] who can more fully consider their circumstances” (Asian woman with self-rated adequate health literacy). “AI is better at playing human games than humans are - it’s already World Go Champion and World Chess Champion...” (Black man with self-reported adequate health literacy)
Human-AI collaboration	39 (13.1)	“While I think that AI will be useful in mental health scenarios, I think that oversight should still be done, just like how I would prefer a few doctors to confirm a diagnosis. I think that it will be good at detecting some trends that can help move people towards better help, but that the help itself should be a joint effort and more personalized” (White nonbinary individual with self-reported adequate health literacy). “Overall, the use of AI is to assist the doctor in providing an accurate diagnosis. It improves the reliability of the diagnosis” (Asian man with self-rated adequate health literacy).
Overreliance on AI	30 (10.1)	“The biggest ethical concern I can think of is a mental health professional being completely reliant on AI without taking a closer look into how the program works” (White woman with self-rated adequate health literacy)
Human reasoning and communication	54 (18.2)	“That it can’t pick up on the subtleties of some symptoms that a human can” (White man with self-rated inadequate health literacy).
Importance of human connection	90 (30.3)	“Human connection and understanding are crucial in mental health diagnosis and treatment. I finally found a doctor that made me feel understood, heard, and cared for. It resulted in an effective treatment for my major depression and suicidal ideology after many years. AI can’t do that” (White woman with self-rated adequate health literacy).
Jobs	6 (2.0)	“As someone who will be working in healthcare in the new future, I am concerned that AI in health scenarios will take away jobs from real people who put in all the work to be working there” (White woman with self-rated adequate health literacy).

^a^Percentages do not add to 100%, as “no additional concerns” and “indeterminable” codes are included in the count.

### Additional Values and Concerns

Respondents expressed further values and concerns beyond performance and human-AI dynamics, many of which were also covered in the closed-ended survey responses, including trust, transparency, privacy, responsibility, and cost (Table 6).

**Table 6:** Detailed qualitative codes related to additional values and concerns regarding the use of artificial intelligence (AI) for mental health.

Detailed code	Count (n=222, 21.2%), n (%)^a^	Example quote
Privacy	98 (44.1)	“I would be concerned about the company that owns the AI and if they could share the data with third parties. I’d also be concerned about what happens if someone admitted suicidal thoughts” (White woman with self-rated adequate health literacy).
Transparency	22 (9.9)	“I would worry about how my data is used to make AI based decisions. I would also wonder about the type of data being used” (White man with self-rated adequate health literacy).
Ethics	23 (10.4)	“One worry is that AI might be used to diagnose and treat mental health conditions without a person’s consent. This could lead to people being treated for conditions they do not have, or not receiving treatment for conditions they do have...” (White man with self-rated inadequate health literacy).
Trust	26 (11.7)	“I am just not comfortable with any machine diagnosing and treating any symptoms of mine, bottom line” (White man with self-rated adequate health literacy).
Appropriate use	19 (8.6)	“Yes, that a manipulative enough person could sway the machine into getting what they want rather than what they need” (White man with self-rated adequate health literacy).
Responsibility	14 (6.3)	“I wonder who would be held liable in the event a patient dies or experiences bad side effects due to the diagnosis or advice of AI. Would it be the AI itself or would the specialist also be held accountable?” (White woman with self-rated adequate health literacy)
Cost	20 (9.0)	“I think if the purpose is to provide better and more thorough health care, then it is a good endeavor. If the purpose is to decrease the costs of providing health care while maximizing profits, the project is specious. That’s why I can see it as a diagnostic tool to help healthcare providers come to a more accurate and thorough diagnosis. But I think it’s about maximizing profits and all stages of the healthcare process” (Asian woman with self-rated adequate health literacy)

^a^Percentages do not add to 100%, as “no additional concerns” and “indeterminable” codes are included in the count.

## Discussion

### Principal Findings

This is one of the first studies to explore public perspectives of the use of AI for mental health–related applications. Our results expand upon other works studying public perceptions of AI for non–mental health care applications and raise important considerations regarding patient involvement in AI use for their mental health [31,44]. We also focused on various applications of AI to mental health care, differentiating our results from previous user-centered design studies that have elicited participant perceptions of a single, specific AI tool under development. Our study highlights the nuances of patients’ perspectives regarding AI for mental health care, revealing that their comfort with AI use depends on the purpose of the AI (tasks it performs), use process (when it is used and what factors drive predictions), and performance of the AI (how well it works and what happens when it is wrong) [45].

### Perceived Benefits of AI for Mental Health Care (RQ 1)

Just under half (245/497, 49.3%) of the respondents in our study reported that they thought AI would make mental health care better or somewhat better. This is similar to a study conducted in Germany where 53% of patients reported positive or very positive attitudes toward AI, but not specifically for mental health [46]. Participants in other studies asking perceptions regarding more specific applications of AI (eg, a radiology image interpretation study in Saudi Arabia and a pregnancy and postpartum exploration in Spain) had stronger positive attitudes toward AI [47,48]. This is consistent with qualitative studies that have found patients with specific health challenges more readily connected with AI’s potential benefits. Our study also found that women were associated with lower perceived benefits of AI for mental health, while lower self-rated health literacy and Black or African American race were each associated with more positive perceptions. The previously cited study conducted in Germany had similar findings related to lower perceived benefit among women [46]. However, it is interesting that this result remained consistent for mental health care applications, in light of the fact that women have reported lower levels of stigma regarding mental health care than men [49,50]. Our study also detected an interesting paradox that those having lower self-reported health literacy had more positive perceptions toward AI for mental health, although this finding warrants further replication and investigation. Regardless of patient perceptions, the inclusivity of patient-facing information regarding AI, ensuring those of various levels of literacy and numeracy may equitably comprehend its functions, remains critically important. Those of African American and Black race in the United States consistently report greater stigma and lower levels of trust toward mental (and other) health institutions due to biases, discrimination, and systemic racism [51,52]. The greater perceived benefit of AI for mental health care may represent a view that AI can be more just and without the biases of humans. This notion also requires further exploration, particularly given that the biases of human can often be embedded into the AI because training data embody previous human behavior.

### Concerns Regarding AI for Mental Health Care (RQ 2)

Participants in our study cited concerns consistent with previous work related to AI accuracy, risk of harm (eg, wrong diagnosis and inappropriate treatment) [44,53,54], decreased human communication and connection [35,44,48,53], and issues pertaining to confidentiality [35,44,48,54-57]. Issues related to privacy were also the most commonly mentioned concern in the qualitative feedback. Participants also qualitatively described concerns about the performance of AI and doubts in AI’s ability to truly replicate human reasoning. In our study, participants expressed some concern related to rising costs, although, as found in other studies, this worry was less pronounced [35,58]. These results continue to stress the importance of contextualization for patients in terms of the following: the accuracy of AI, harms and how they are mitigated, and data use and protections. As described in previous studies, continuing to support human connection is particularly needed in mental health care applications given the importance of the patient–mental health care professional therapeutic relationship.

### Comfort With AI Accomplishing Mental Health Care Tasks (RQ 3)

Our study provided further evidence that patients’ comfort with AI varies based on what the AI does. People were the least comfortable with diagnosis delivery tasks, which lends further support to the importance of continuing to keep health professionals in the loop related to AI [35]. Similar to a previous study of pregnant people in Spain [47], patients were most comfortable with tasks that recommended general wellness strategies or talk therapy. These results also suggest that AI for tasks patients are less comfortable with may require greater care to explain and also emphasize how the AI works with the health professional.

Despite the rapid proliferation of chatbots [37,59], less than half of the participants (237/500, 47.4%) were comfortable sharing mental health information with a chatbot, which may simply signify that these types of tools should be used on an opt-in basis. Previous studies have suggested that it may be easier for someone to share these sensitive feelings with a computer or AI [60], and this may be true for certain people, but our findings did not universally support this assertion. It was also notable that approximately a quarter of the sample was not comfortable sharing mental health information with a mental health care professional. We acknowledge this may have been impacted by the types of mental health information listed in our survey, but it may also represent a continued stigma related to sharing mental health concerns.

### Values Related to AI for Mental Health Care (RQ 4)

Findings in our study related to patients’ values for AI for mental health care revealed challenges that AI integration may present to the patient–health professional relationship. In individual scenarios, patients overwhelmingly found mental health care professionals responsible for AI-related errors. While this does reflect similarity to the current standard of practice (ie, that health professionals are held responsible for medical errors even when computer systems are involved), future work should consider how this affects health professional well-being given the challenges related to burnout and shortages in trained mental health care workers. It also supports programs, such as AI programs falling under the US Food and Drug Administration’s purview and the European Union’s AI Act of 2023, where algorithms may be reviewed before use and subject to regulations [61]. Participants also noted that their trust in their mental health care professional would decrease if their assessment disagreed with AI. However, this was somewhat at odds with relatively fewer patients viewing issues with AI decreasing trust in their mental health care professional as “very important.” It was also notable that approximately one-third (137/500, 27.4%) of participants said they “did not know” how this scenario would affect their trust in their mental health care professional, which seems to highlight that patients may still be wrapping their heads around feelings regarding emerging technologies, such as AI.

Patients desired a high degree of transparency related to AI use, with >90% of participants considering it important that they be told when AI played even a small role in their care. Participants also valued explainability, as most participants (289/500, 57.8%) were not comfortable with highly accurate AI that could not explain how it made its predictions and as “understanding individual risk factors” was rated as the most important value related to AI for mental health care applications. It is at best unclear what patients are typically told regarding when AI is used for their care; how explainable it is; and to what extent, if at all, they are informed what factors drive predictions regarding their care. These results suggest that patient values may be at odds with the current standard of practice for patient communication. Similar to the concerns previously described, participants also highly rated the importance of AI not leading to errors and helping with their mental health symptoms.

### Implications and Future Challenges

Implications regarding our findings are organized topically and include concrete recommendations, which have been italicized for emphasis.

#### Navigating the Patient–Health Professional Relationship

The therapeutic relationship between a patient and their health professional is crucial in mental health care settings. Our study revealed issues that will need to be reconciled if AI is to be safely, transparently, and acceptably used for mental health care. From our results, it is clear that patients want mental health care professionals to be the ultimate decision makers, using AI to support (but not make) decisions when it is deemed safe and effective [36]. Participants also overwhelmingly viewed mental health care professionals as the people responsible if an error occurred related to treatment where AI had been used.

Future work should investigate shared regulations for AI responsibility, health professional competencies for AI use (Russell et al [62]), and interfaces that support shared decision-making when using AI.

Specifically, designs should support collaborative patient–health professional decision-making in a way that fosters trust instead of degrading it while also not creating undue burden for the health professional. Previous studies have described how clinicians should be able to contest AI, such as ignoring it (when it is not relevant or appropriate), trusting it when it is appropriate, or being able to uncover explanations to negotiate in borderline cases [63]. Such systems should be able to track health professional decisions in relation to the AI, possibly allowing health professionals to provide brief rationale that they may use in conversations with patients. In creating such systems, usability and model explainability will be critical. While these systems may be difficult to study in situ given the sensitivity of mental health conversations, *solutions may first be evaluated in realistic clinical simulation environments to ensure safety and usability prior to larger scale deployment.*

#### Communicating AI-Related Information to Patients

Participants in this study desired various information regarding the use of AI for their mental health care; that is, they wished to know when, for what, and why it was used; how accurate it was; and the risk factors that drove a decision. Even with highly predictive AI, patients were still not comfortable with AI that could not explain how it arrived at a result, and they qualitatively expressed concerns related to misdiagnosis and improper treatment that could result from AI use. They also reported “understanding individual risk factors” as the most important value. It is unclear as to how much if any “information” patient receive, and there seems to be a mismatch between the current deployment of AI for mental health care and patients’ desires. *At minimum, we recommend promoting transparency in AI’s use, the communication of its accuracy, and including individual risk factors to help patients and clinicians decide when AI may be appropriate for use in health-related applications. Communications should also address how potential biases (eg, in the training data) have been evaluated and mitigated in the resultant* AI *tool.*

Communicating the desired information to patients, however, is not straightforward, as concepts such as AI performance and process involve complex mathematical concepts. Furthermore, this desire for additional information regarding AI is also at odds with how patient communication has traditionally been practiced. When we consider other non–AI-based diagnostic or decision support tools (eg, magnetic resonance imaging, blood tests, and screening assessments), communicating information regarding how they work (ie, their process) or their performance is far from standard practice. AI seems to be held to a higher standard than other diagnostic tools related to transparency in performance. *Future work should consider not only communicating the process and performance of AI but also providing this information in the context with the performance of the existing approach to a given task.* This would allow health professionals and patients to determine whether the potential benefits of AI outweigh their concerns.

Providing patients with explanations of AI performance and factors driving prediction will require extensive study involving experts in human-centered, inclusive design working alongside AI developers, mental health care professionals, and patients. *There is a need for laboratory-based studies to understand what information regarding AI balances recognizing patient values, but also supports comprehension of important concepts and fostering appropriate trust.* These issues raise many questions for numeracy and data visualization experts regarding how information may be conveyed inclusively to patients with different needs so that the benefits of AI may be equitably realized.

#### Individuality and Autonomy

Our study detected differences in who may find AI beneficial and for what tasks they may be comfortable using it. *Future work should explore how we may respect individual autonomy with regard to the use of AI applications so that patients and health professionals may collaboratively make decisions about the appropriate application of AI to mental health care issues.*

### Limitations

Our study was limited in that the sample was recruited using a web-based platform, which may not generalize to those with technology, literacy, and other barriers to web-based survey completion. The sample was representative of the adult US population in terms of age, gender, and race distributions (Prolific/academic researchers), leading to most of the survey respondents being White due to >70% due to the US demographic makeup (US Census Bureau [64]). Therefore, important perspectives from other racial and ethnic groups may be limited. Our results may also be subject to response bias, as those who had strong feelings regarding topics pertaining to AI, mental health, or their intersection may have been more likely to respond. For example, a higher-than-anticipated proportion of respondents (215/500, 43%) reported having a history of mental illness. We did attempt to control for this in RQ 1, and the history of mental illness was not found to significantly affect responses regarding participants’ perceived benefits of AI for mental health care. We, however, did not have a mechanism for evaluating how preexisting attitudes of AI may have affected responses, and this may be addressed with validated measures of AI attitudes in future surveys (Schepman and Rodway [65]). Given the scope of this paper, we were unable to assess sociodemographic differences (eg, based on race and literacy) across all outcomes. Future work may use this or other data sets to provide a more nuanced picture of differing views related to specific questions regarding AI for mental health.

### Conclusions

Our study found that approximately half (245/497, 49.3%) of the US adults surveyed perceived some benefit for the use of AI in mental health care applications. These perceived benefits were lower among women but higher among Black or African American participants and those with lower self-rated health literacy. Participants also expressed nuanced differences in the types of tasks they would be comfortable with AI completing, showing the greatest discomfort with AI handling clinical diagnosis, diagnosis delivery, and the recommendation of medication. Those surveyed valued high-performing AI that could explain individual risk factors driving predictions. In general, participants were concerned that AI may mean a loss of human connection, and they perceived humans as the ultimate decision makers, with AI serving as an additional data point when appropriate. Qualitative feedback also revealed participants’ deep-seated fears regarding the use of AI for their mental health care. These findings stress the importance of working with patients and mental health care professionals to understand whether and how AI may be safely, ethically, and acceptably implemented for mental health care applications.

## Appendix

Multimedia Appendix 1 Full survey battery, including exact questions and answers.

## Abbreviations

AI: artificial intelligence
OR: odds ratio
RQ: research question

## Data Availability

Data are available upon reasonable request to the corresponding author.
